# Decision-making in the detection and management of patients with sepsis in resource-limited settings: the importance of clinical examination

**DOI:** 10.1186/s13054-018-1971-7

**Published:** 2018-03-01

**Authors:** Rashan Haniffa, Abigail Beane, Arjen M. Dondorp

**Affiliations:** 1 Network for Improving Critical Care Systems and Training, 2nd Floor, YMBA Building, Colombo, 00800 Sri Lanka; 2 Mahidol Oxford Tropical Medicine Research Unit, Faculty of Tropical Medicine, Mahidol University, Bangkok, Thailand; 3 Centre for Tropical Medicine, Nuffield Department of Medicine, University of Oxford, Oxford, UK

We read with interest the study by Andrews et al. [1] and the related correspondence from Shrestha et al. [2]. We share the concern that clinical examination (and observations) appear(s) to be perceived as relatively unimportant in both the detection of the unwell patient and in the titration of interventions such as fluids, oxygen, antibiotics and vasopressors in LMICs. Studies have highlighted the limited availability of clinical observations in acutely unwell LMIC settings, which in addition to hindering detection of the deteriorating patient also complicates evaluation of an individual’s treatment and standards of care evaluation; for example, the assessment and implementation of early warning scores and prognostic models [3]. In settings where potential for rescue by resource-intense interventions (e.g. ventilation) is remote, we too are surprised by the absence of a more central role for clinical examination and observations. It is of further concern that such limitations remain in the relatively high-resource, high-visibility environment of a clinical trial.

These findings raise questions regarding our understanding of the decision-making process of LMIC clinicians in the detection and management of the acutely unwell patient. Clinicians may be utilising additional cues in a manner different to their HIC contemporaries; such as the presence of relatives as carers at the bedside or a nursing decision to place a patient in a specific location in the ward. Work done to evaluate the impact of setting-adapted practical training on the management of common emergencies in LMICs has highlighted the limited priority given to practising such skills in existing training programmes for both doctors and nurses [4, 5]. It is also possible that clinical examination and vital signs measurements are performed but poorly recorded: in part a consequence of disparate paper-based records.

Greater understanding is essential if we are to better influence the processes that contribute to acute and critical care mortality in LMIC settings. Mixed-method approaches combining qualitative techniques to capture clinicians’ perceptions of the importance of clinical assessment alongside setting-adapted electronic tools to improve the capture of granular information of the patient journey—currently being undertaken by our group in multiple LMIC settings—could enrich our understanding of the management of acutely unwell patients. Greater understanding of these clinical priorities and their importance in acute care in diverse settings would offer valuable insights to inform subsequent trial design and the end points selected for evaluation.

## References

[1] Andrews B, Semler MW, Muchemwa L, Kelly P, Lakhi S, Heimburger DC, Mabula C, Bwalya M, Bernard GR (2017). Effect of an early resuscitation protocol on in-hospital mortality among adults with sepsis and hypotension: a randomized clinical trial. JAMA.

[2] Shrestha GS, Dünser M, Mer M (2017). The forgotten value of the clinical examination to individualize and guide fluid resuscitation in patients with sepsis. Crit Care..

[3] Beane A, De Silva AP, De Silva N, Jayasingha AS, Rathnayake DRM, Sigera PC, Athapattu PL, Mahipala PG, Rashan A, Munasinghe SB, Jayasinghe KSA, Dondorp AM, Haniffa. Evaluation of the feasibility and performance of existing Early Warning Score (EWS) to identify patients at risk of adverse outcomes in a low-middle income country (LMIC) setting: a longitudinal observational cohort study. BMJ Open. Accepted for publication January 2018. Manuscript ID:2017–019387. in press 10.1136/bmjopen-2017-019387 PMC5922475 29703852

[4] Haniffa R, Lubell Y, Cooper BS, Mohanty S, Alam S, Karki A, Pattnaik R, Maswood A, Haque R, Pangeni R, Schultz MJ (2017). Impact of a structured ICU training programme in resource-limited settings in Asia. PLoS One..

[5] Beane A, Padeniya A, De Silva AP, Stephens T, De Alwis S, Mahipala PG (2017). Closing the theory to practice gap for newly qualified doctors: evaluation of a peer-delivered practical skills training course for newly qualified doctors in preparation for clinical practice. Postgraduate Medical Journal.

